# Some Interesting Experiments in Physical Efficiency

**Published:** 1920-07-24

**Authors:** 


### BLOOD PRESSURE AND FATIGUE.

### Some Interesting Experiments in Physical Efficiency.

ALTHOUGH not primarily devoted to the study of fatigue in general the small book "Air Sickness: its Nature and Treatment,"* recently published by Cruchet and Moulinier, contains a particularly interesting description of a theory of physical exhaustion, which can be applied equally well to the study of the ætiology of air-sickness and to a number of other occupational ills. The whole book is remarkably refreshing in its manner of bringing scientific facts into close relation to everyday life, but in this short note we will confine our attention to that section whereon the authors base their chief deductive reliance.

### A COMPARISON.

There are many people who believe that the factor of fatigue, mental as well as physical, is of fundamental importance in a host of economic problems of the day. As far as airmen are concerned, we may liken the disturbances occurring in their constitutions to those experienced by athletes in the course of various tests of endurance, such as horse-riding, motor-racing, cycling, flat-racing, etc. Apparently the first sign of athletic fatigue, as far as can be experimentally ascertained, is an arterial pressure fall, but it is, in actual fact, far harder in this way to estimate fatigue than is frequently supposed. Many people who complain of being tired are less exhausted than they say, while on the other hand, there are many who are in reality far more exhausted than their own senses suggest. In fact fatigue is largely a subjective sensation which we try to express in phrases which are neither precise nor capable of trustworthy comparison. Similarly, we can comment upon fatigue as a pain, in that it is a quality which varies with each individual, and that so long as we confine our attention to the information provided by the subject himself, we cannot possibly measure the physiological state in true mathematical terms. One man will complain that he is completely knocked up, when any careful examination proves him to be very far from collapse, whereas many an athlete after a hard game or a long run will protest that he is as fresh at the end as he was at the beginning, whereas his heart sounds, his pulse rate and the record of his arterial pressure enable us to diagnose indisputable fatigue.

Therefore we must attempt to find scientific methods which eliminate as far as possible the personal factor. In the past we have turned successively to the examinations of cardiac and other muscles or to the minute study of the results of urine analysis. But none of these past measures proved satisfactory either to explain all the mechanism of fatigue or to enable us to assign to each individual the quantity of work he might be justifiably expected to do. Dr. Pachon, in a notable memoir in 1910, solved the problem in the following way. It was known that the distress of a man in bad training is due to reactions associated with the heart, and that it is in this quarter that pathological results are likely to follow an ill-considered strain. The question then was how to track the origin of this reaction of cardiac stress, and the investigator chose two subjects, the one in training for a life of physical activity, the other untrained and accustomed to a sedentary existence. They were put through the same exercises, and it was found that in the case of the latter the values (maxima and minima) of arterial pressure were both immediately diminished, whilst in the trained individual the values not only showed no sign of fall, but were, indeed, slightly increased. In short, it was a test of a person's power of adaptation—or more accurately the working value of his cardiac system.

Let us now see what happens to the untrained man when fatigue begins to make itself felt. In the first instance, as the exercise commences the pressures rise up to the working level, but sooner or later, according to the onset of fatigue, there is a fall, chiefly of the maxima, indicating a definite failure of the heart to adapt itself to exertion. This initial diminution of maximum blood-pressure was described by Professor Pachon by the expressive phrase of the "danger signal." He proved conclusively that in practice this phenomenon immediately precedes the moment when fatigue is shown in an objective manner, whether or not it is perceived subjectively at the same time. If exertion be continued the individual overworks his heart, and the pressure falls still lower, until a stage is reached in which, when the exercise ceases, the pressure may actually rise again, in spite of the state of complete and obvious fatigue. Generally, however, in exhaustive states lower pressure not only appears at the time of the greatest strain, but it continues for a greater or less period during subsequent rest.

### THE ORIGIN OF EXHAUSTION.

Thus, to recapitulate, all work, all output of energy, is accompanied by changes in the circulation. When the work done does not exceed the limits of physiological resistance—either natural or acquired by training—the observed variations of arterial pressure follow a rhythm which may be considered as the criteria of the patient's adaptation to that work. We can readily follow the pressure changes of individuals in most forms of exertion, and the originator of these observations has claimed to be able thereby to keep absolutely reliable control over the training of athletes. But, turning specifically to aviation, we must remember that there are some exceptional differences in the types of strain to which the airman is subjected. Without discussing the details of these, it may be noted that the minimum pressure decreases during ascent in inverse ratio to the altitude, and rises again during descent, this rise being more marked as the

* "Air Sickness: its Nature and Treatment." By Cruchet and Moulinier. (John Bale, Sons and Danielsson, Ltd., London.)

**Blood Pressure and Fatigue**—(*continued*).

speed of the descent becomes more rapid. This rise of the minimum blood-pressure is a phenomenon peculiar to aviation, and it is a complete contradiction to the phenomenon which we have described as occurring in other tests of endurance. The point that the authors make is that this anomaly is directly due to the changing atmospheric pressure, and although in practice it appears to vitiate the accurate study of the cardiac muscle under aviation conditions, yet it is of the greatest importance as an observation to prove the enormous extra strain set upon the airman's heart if the air pressure continually rises and falls.

We have selected but a small section of this valuable little work, and strongly advise those interested in medical work of this kind to consult its pages, whether their interest in the vagaries of cardiac strain results directly from experiences in the flying world or simply in occupational strain.

